# Assessing core competences of medical students with a test for flight school applicants

**DOI:** 10.1186/s12909-018-1438-1

**Published:** 2019-01-07

**Authors:** Sigrid Harendza, Henning Soll, Sarah Prediger, Martina Kadmon, Pascal O. Berberat, Viktor Oubaid

**Affiliations:** 1 III. Department of Internal Medicine, University Medical Center Hamburg-Eppendorf, Hamburg, Germany; 2 III. Medizinische Klinik, Universitätsklinikum Hamburg-Eppendorf, Martinistr. 52, D-20246 Hamburg, Germany; 3 German Aerospace Center (DLR), Hamburg, Germany; 4 Medical Faculty, Deanery, University of Augsburg, Augsburg, Germany; 5 TUM Medical Education Center, School of Medicine, Technical University of Munich, Munich, Germany

**Keywords:** Assessment, Competences, Flight school applicants, Professionalism, Undergraduate medical education

## Abstract

**Background:**

Important competences of physicians regarding patient safety include communication, leadership, stress resistance, adherence to procedures, awareness, and teamwork. Similarly, while selected, prospective flight school applicants are tested for the same set of skills. The aim of our study was to assess these core competences in advanced undergraduate medical students from different medical schools.

**Methods:**

In 2017, 67 medical students (year 5 and 6) from the universities of Hamburg, Oldenburg, and TU Munich, Germany, participated in the verified Group Assessment Performance (GAP)-Test at the German Aerospace Center (DLR) in Hamburg. All participants were rated by DLR assessment observers with a set of empirically derived behavioural checklists. This lists consisted of 6-point rating scales (1: very low occurrence to 6: very high occurrence) and included the competences leadership, teamwork, stress resistance, communication, awareness, and adherence to procedures. Medical students’ scores were compared with the results of 117 admitted flight school applicants.

**Results:**

Medical students showed significantly higher scores than admitted flight school applicants for adherence to procedures (*p* < .001, d = .63) and communication (*p* < .01, d = .62). They reached significantly lower ratings for teamwork (*p* < .001, d = .77), stress resistance (*p* < 0.001, d = .70), and awareness (*p* < .001, d = 1.31). Students in semester 10 showed significantly (*p* < .02, d = .58) higher scores in domain awareness compared to the final year students. On average, flight school entrance level was not reached by either group for this domain.

**Conclusions:**

Advanced medical students’ low results for awareness are alarming as awareness is essential and integrative for clinical reasoning and patient safety. Further studies should elucidate and discuss whether awareness needs to be included in medical student selection or integrated into the curriculum in training units.

## Background

The measurement of competences, abilities, and personality dimensions has a long tradition in psychology in order to predict future behaviour, educational, and occupational success. Today, psychological tests and assessment methods are well-used in several industries worldwide to train or hire the right stuff. Competences play an increasingly important role in undergraduate medical education. Especially social-interactive skills like interprofessional communication are essential for medical students’ development of professional behaviour [1]. The integration of different competences will eventually lead to the mastery of professional medical activities [2]. Workplace-based assessments are developed longitudinally to measure students’ performance with respect to the development of these competences [3, 4]. Based on a pilot study [5], we developed a 360-degree assessment to test the core competences of advanced undergraduate medical students during a simulation of their first day in the role of a resident in a hospital [6]. This assessment used methods typically applied in assessment centers [7] and included a consultation hour with five simulated patients, a patient management time with interprofessional interactions, and a patient handover. It was based on the competences teachers from the three medical schools with different types of undergraduate curricula rated to be of particular relevance for a beginning resident [8]. Whether this assessment correctly measures the respective competences required for patient safety had to be assessed for construct validity.

The main use and objective of assessment centers is to predict future job performance in current or perspective personnel within a professional entity. These predictions are made by testing a specific set of competences. Therefore, the better competences tested in an assessment center correspond to the demands of the respective profession, the better the prognostic success of an assessment center [9]. In medical education, some research explores the effects of competence-based assessment centers for medical school admission, which includes tasks like studying information, answering questions regarding the information and explaining aspects of the studied information [10–12]. How final year medical students or physicians would perform in such assessments is not known. When physicians from five specialties were asked to evaluate core competences relevant for their profession they ranked stress resistance, leadership, adherence to procedures, teamwork, and communication as the five most relevant core competences [13]. The rating process was carried out via Trait-Tracker© [14], an instrument for rating core competences with occupational relevance. The core competences adherence to procedures and stress resistance received the highest ratings from surgeons [14]. Similar to the job specification for surgeons, the requirement profile for airplane pilots also includes cognitive and interpersonal competences besides expertise [15].

Flight school applicants, who wish to become airplane pilots, have to successfully complete an assessment, which includes the assessment of core competences besides the assessment of knowledge and skills [16]. Since the respective core competences included in the assessment center for flight school applicants match the main core competences required for physicians, we intended to elucidate how advanced undergraduate medical students are assessed with respect to these core competences when performing the flight school application assessment center tasks.

## Methods

### Group assessment of performance (GAP)-test for flight school applicants

The test program for flight school applicants includes cognitive ability tests, work sample tests, and an assessment center. The assessment center is a method to evaluate social and interactive skills in applicants and a meta-analysis on assessment center validity shows significant contributions to the prediction of occupational success [17]. The DLR assessment center includes a role play and the group task GAP, and finally a semi-structured interview. The group task GAP assesses the candidate’s competences to co-operate and work under time pressure in team environments. It was chosen for the present study and includes the following domains of competence: leadership (LS), teamwork (TW), communication (CM), stress resistance (SR), awareness (AW), and adherence to procedures (AP). The domains and their facets are described in Table 1.

**Table 1:** Domains of competences including their definitions and facets

Domain	Definition	Facets
Leadership (LS)	Good leadership is defined by specifying goals and controlling and correcting the outcome. A good leader considers the situations of others. It includes decision making, the ability to take the appropriate actions after having assessed the risks and benefits of several options.	▪ Guidance/commandability ▪ Assertiveness/persistance ▪ Decision making ▪ Resistance to premature judgement ▪ Analytical methods ▪ Finding solutions actively
Teamwork (TW)	Good teamwork consists of active participation and co-operation in group processes. Good team members share their information and ask others about their views and ideas. It involves a sensitive perception of social situations and a tolerant and respectful treatment of others’ intentions and actions. This implies to perform tasks and responsibilities conscientiously and trustworthy.	▪ Social sensitivity/empathy ▪ Sociability ▪ Tolerance ▪ Honesty ▪ Active helpfulness
Stress resistance (SR)	Maintaining effective performance, control and goal orientation under pressure or adversity. A good stress resistance includes also the absence of physiological symptoms (vegetative, motoric or verbal).	▪ Resilience ▪ Stress resistance ▪ Strain symptoms
Awareness (AW)	Awareness is the ability to remain always cognizant of the surroundings. It involves recognizing how information, rules, own actions and actions of others influence the present and future. Cognitive resources are limited. The discrepancy between situational demands and the individual possibility to perform accurately accounts for problems when fulfilling a task.	▪ Situational awareness ▪ Workload management
Communication (CM)	Communication includes information transfer and social aspects. Crew members share information and assure reception and understanding. Suggestions of other crew members are considered, even if one does not agree. Ambiguities and uncertainties are announced.	▪ Oral fact finding ▪ Persuasion ▪ Oral defense
Adherence to procedures (AP)	This competence is defined by knowledge and disciplined and correct application of rules.	▪ Knowledge of procedures ▪ Adherence to procedures

The GAP-test is a computerized team task, where up to four applicants are in the role of flight attendants [17], sitting at their own workstation. It consists of two subtasks: a planning task and a conflict management task. In the planning task (10 min), the candidates have to plan a number of flights for Christmas and the New Year. It is followed by a conflict management task (8 min), where the candidates have to find a solution for diverging interests in the team. The teams were randomly assembled into groups of three or four participants. Each team received a standardized verbal and written test instruction. The whole GAP-test took about 1.5 h. The GAP test has already demonstrated its psychometric qualities [18], e.g. significant reliability of observer ratings and validity of GAP results: the selection results of flight school applicants (positive vs. negative selection result) showed corresponding mean differences in the independent GAP measures.

One DLR aviation psychologist with more than 15 years and 2000 cases of experience observed a group of candidates. The participating DLR aviation psychologists passed a one-day standardization seminar prior to the assessment. The observer uses a set of empirically derived behaviour checklists [18] for each competence presented on a touch screen, e.g. “strategic suggestion” (positive behaviour for leadership) or “interrupts others” (negative for teamwork) with a 6-point scale (1: very low occurrence to 6: very high occurrence). The inquiry of the assessment of adherence to procedure, for example, is: 1: adheres repeatedly not to several rules, 2: adheres repeatedly not to one rule/adheres once not to several rules, 3: adheres once not to one rule, 4: adheres to all rules, 5: adheres to all rules and gives references to the rules, 6: adheres to all rules, gives references to the rules and corrects team-colleagues’ non-adherence to the rules.

### Participants

The 70 undergraduate medical students, who participated in the 360-degree assessment of a simulated first day in hospital for a beginning resident in July 2017 [6], also completed the GAP-test for flight school applicants in July 2017 at the German Aerospace Center (DLR) in Hamburg. All students were included in this study on a first come, first serve basis and were not randomly chosen for participation. Three students had not reached semester 10 and were excluded from the statistical analysis. Of the 67 advanced undergraduate medical students 53.7% were female and 61.2% were in their final year of a six-year (i.e. 12 semesters) undergraduate medical training program. Of the 408 flight school test applicants (16.9% female) in 2013, 117 applicants, who passed the test (15.8% female), were included in the study. The flight school applicant sample had to be drawn from 2013, because no larger recruitment campaigns have been carried out in the following years. The scores of the selected flight school applicants were used for further statistical analysis.

### Statistical analysis

The statistical analysis was performed on SPSS Statistics 21. Mean scores and standard deviations were computed for all subgroups. For the comparison of subgroup mean scores, t-tests for independent sample were performed and effect sizes (Cohen’s d) were determined. A *p*-value < .05 was considered significant.

## Results

**Fig. 1:**
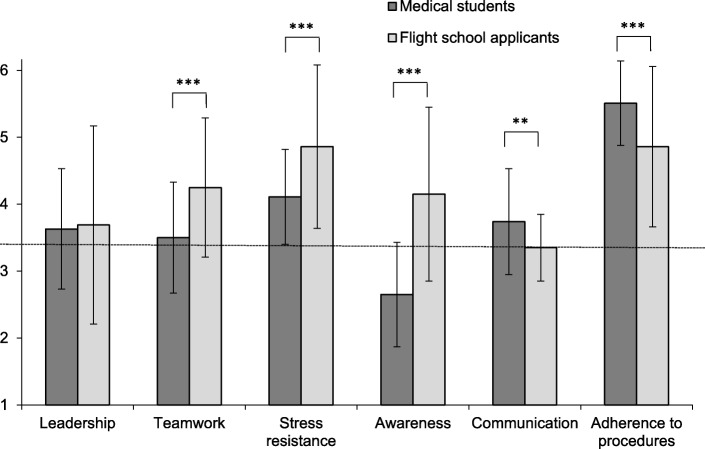
Comparison of scores in six core competences between advanced undergraduate medical students (*n* = 67) and selected flight school applicants (*n* = 117); **: *p* < .01, ***: *p* < 0.001; dotted line marks the grade point average required for entrance to flight school

**Fig. 2:**
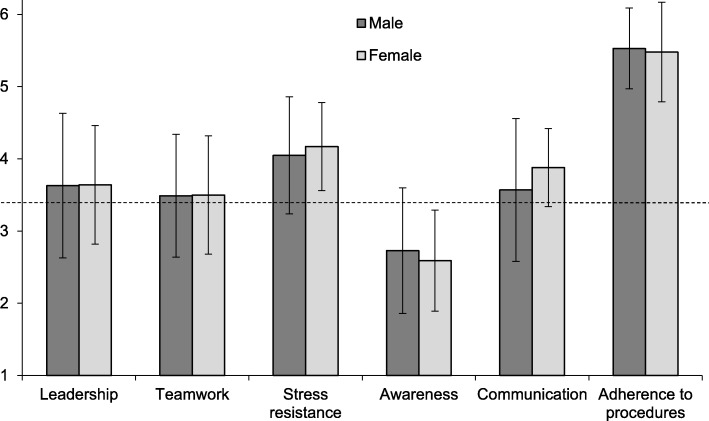
Comparison of scores in six core competences between male (*n* = 31) and female (*n* = 36) advanced undergraduate medical students; dotted line marks the grade point average required for entrance to flight school

**Fig. 3:**
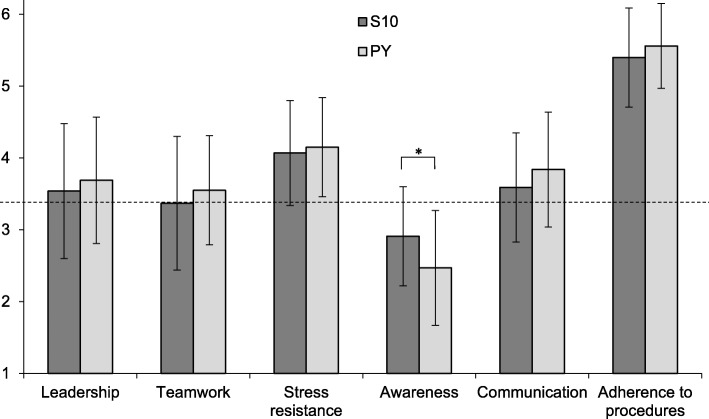
Comparison of scores in six core competences between medical students in semester 10 (S10, *n* = 28) and final year (PY, *n* = 39), *: *p* < .02; dotted line marks the grade point average required for entrance to flight school

**Fig. 4:**
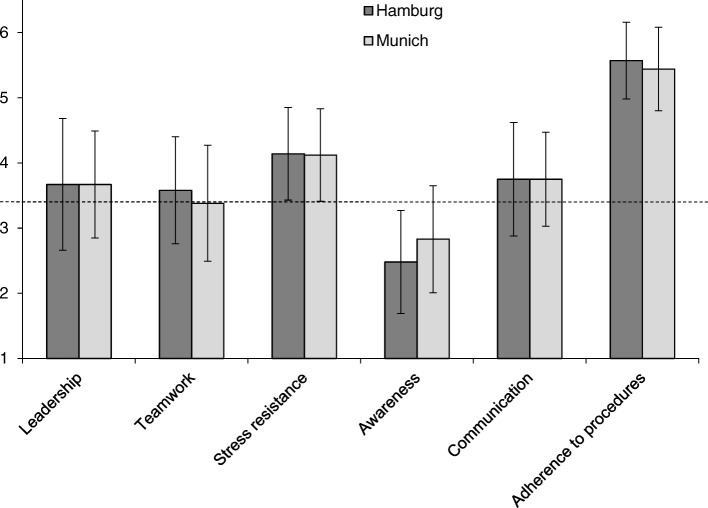
Comparison of scores in six core competences between students from Hamburg (VI curriculum, *n* = 35) and students from Munich (non-VI curriculum, *n* = 26); dotted line marks the grade point average required for entrance to flight school

Advanced medical students (*N* = 67) showed significantly higher ratings than selected flight school applicants (*N* = 117) for the competence domains adherence to procedures (AP), *p* < .001 (d = .63), and communication (CO), *p* < .01 (d = .62), while they reached significantly lower ratings for the competence domains teamwork (TW), *p* < .001 (d = .77), stress resistance (SR), *p* < .001 (d = .70), and awareness (AW), p < .001 (d = 1.31) (Fig. 1). In the competence domain awareness (AW), advanced medical students did not reach the entrance level required for flight school applicants. When the participating female and male medical students were compared, we found no significant difference in any of the six domains (Fig. 2). Students in semester 10 (S10) showed significantly (*p* < .02; d = .58) higher scores in the domain awareness (AW) compared with students in their final year (PY). On average, neither group reached the flight school entrance level in this domain (Fig. 3). No significant differences in any of the domains were found when undergraduate medical students from the University of Hamburg, who study in a vertically-integrated (VI) curriculum (basic and clinical sciences are studied together longitudinally), were compared to undergraduate medical students from the Technical University of Munich, who follow a non-vertically integrated (non-VI) curriculum (basic sciences are studied during the first two years and the studies of clinical sciences begin in year three) (Fig. 4).

## Discussion

Medical doctors and airplane pilots require a similar set of core competences for their respective professional behaviour [13]. We discovered significant differences in the expression of core competences between advanced medical students and accepted flight school applicants. The most striking result with a high effect size was seen in the competence awareness, where medical students showed significantly lower scores – even below the acceptance level for flight school – compared to accepted flight school applicants. Interestingly, medical students in semester 10 (year 5 of a six-year undergraduate medical curriculum) showed significantly higher scores for awareness than final year students with a medium effect but also below the acceptance level for flight school applicants. Awareness is an important core competence for physicians to perform clinical reasoning [19] and to reduce surgical errors in the operating room [20]. In a systematic review on competences to enhance patient safety, awareness emerged as one of the five key themes [21]. Therefore, the low level and particularly the even lower level of awareness we observed in the further advanced medical students seems especially alarming. The difference between the two cohorts may suggest, that medical students not only have a relatively low level of awareness in general, but they might even lose some awareness competence over the time of their undergraduate education. For other competences, e.g. professionalism, a decline of professional attitude has been demonstrated for medical students while they progress through medical school [22]. However, further work is needed to discern whether an actual decline of awareness occurs during undergraduate medical training or not.

As we did not observe differences between students from a VI and non-VI curriculum, the form of undergraduate medical training may not be associated with the decreasing awareness. However, assessment during undergraduate medical education mostly includes multiple choice knowledge tests and objective structured clinical examinations (OSCE) for specific skills. Both usually require little awareness but rather high adherence to procedures. More advanced medical students have shown high levels of adherence to and knowledge of procedures, providing more correct answers in a formative multiple choice exam than less advanced medical students [23]. Even in high stakes multiple choice exams it is very difficult to design questions beyond the clinical reasoning aspect of pattern recognition [24], and medical students provide very little analytical reasoning and much test-taking behaviour while answering multiple choice questions [25]. As far as OSCE performance is concerned, final year medical students performed weakest when clinical reasoning skills were assessed, which require high levels of awareness, and strongest in procedural skills, which require high levels of adherence to procedures [26]. Furthermore, the students of our study, who also participated in the 360-degree assessment of a simulated first day of residency [6], perceived the highest strain during the patient management phase where they had to handle and prioritize different tasks requiring a high level of awareness [27]. Taken together, certain clues from the literature point towards the hypothesis that learning and assessment during undergraduate medical training might not promote awareness competence or even lead towards a reduction of awareness even though awareness is a strongly needed competence for professional performance in clinical situations. On the other hand, awareness can be improved in the context of medical education by game-based training [28] and simulation [29] and can be taught as early as in the first year of undergraduate medical training [30].

Awareness is an important competence with respect to patient safety [13, 14]. Even though the medical students in our study scored very low in this domain on average, individual participants showed very high scores for awareness. This made us wonder whether awareness might be a competence, which can be trained at medical school only to a certain extent and, therefore, constitutes a competence, which should rather be assessed in a selection test for medical school applicants. It is known from postgraduate medical education, that a situational judgement test, which requires awareness as a basic competence, was the best single predictor of performance in a selection center for postgraduate medical education [31]. For the selection into general practice training, a situational judgement test worked equally well to predict performance in workplace-based selection center exercises [32]. For selecting undergraduate medical students, an integrity-based situational judgement test showed psychometric robustness [33]. Hence, the design of situational judgement tests, which requires awareness to find the optimal solution, might be a helpful tool to select students for undergraduate medical education with high levels of awareness.

The mean differences in teamwork and stress resistance (flight school applicants score higher) as well as in adherence to procedures (medical students score higher) might be due to self-selective effects. It is well-known to flight school applicants, that the position of a commercial airline pilot always involves teamwork and, because of its time-critical character, requires a high level of stress resistance. Less cooperative and emotionally unstable persons might be less attracted by a flight school program and therefore not apply. Furthermore, medical students showed the highest level of strain during the patient management phase of a simulated first day of residency, which required a high amount of teamwork [27]. In addition to selecting flight school applicants with respect to high scores for teamwork and stress resistance, crew resource management (CRM) trainings to improve non-technical skills have to be attended by flight personnel throughout their professional career. This may be an indicator that medical schools should focus more on these competences in their undergraduate curriculum. Adherence to procedures, where medical students scored much higher than flight school applicants, is part of the personality trait conscientiousness, which can be measured with the Neuroticism-Extraversion-Openness Five-Factor Inventory (NEO-FFI). The medical students participating in our study show higher scores for conscientiousness in the NEO-FFI than the norm sample (data not shown). Furthermore, medical students demonstrating high levels of conscientiousness were more frequently selected for medical school through a knowledge-based exam [34] and undergraduate medical students with low scores on conscientiousness were less likely to sit their examinations successfully [35]. A literature review confirmed that in general conscientiousness was found to be a significant predictor of performance in medical school [36]. This relationship between conscientiousness and performance at medical school was even shown to become increasingly significant with the advancement of students through medical training [36]. This might explain the high scores for adherence to procedures of the quite advanced medical students in our study. The higher scores medical students reached for communication compared with selected flight school applicants might reflect the effects of communication training medical students receive during their undergraduate studies while most flight school applicants have just finished high school.

We had expected that medical students would score fairly high towards the end of their undergraduate studies in all six domains of the GAP-test, because these six competences are all required for their future work as residents to provide safe patient care. As there seems to be room for improvement – except for adherence to procedures, which is well developed – some aspects, e.g. teamwork or leadership might require a stronger focus in undergraduate medical education and some aspects, e.g. awareness, even need to be addressed in the selection process for medical school.

### Strengths and weaknesses of this study

A strength of our study is that the flight school applicants and the medical students have both undergone a selection process. However, it is a weakness of our study that the flight school applicants are at the beginning of their education and the medical students advanced at least five years into their undergraduate studies, which potentially signifies a developmental difference. Another weakness of our study is the prominence of male selected flight school applicants versus the prominence of female participants in the group of medical students. However, no gender difference was found in the occurrence of the six competences in the GAP-test between female and male medical students. The participating medical students were not randomly chosen, but selected on a first-come, first-served basis after an email announcement at the three universities inviting all students from semester 10 and higher. This might have led to the application of only motivated students or students with good grades. If this were the case, the low values for awareness would be even more intriguing. Despite these limitations our findings seem to be significant with respect to undergraduate medical curriculum design and medical student selection. To answer the question whether awareness is low from the beginning of undergraduate studies or decreases during undergraduate medical education and in order to be absolutely comparable to pre-flight school students, the study would have to be repeated with medical students who have newly been selected for medical school. Furthermore, medical educators should be aware that it might be relevant for medical education like for flight school that certain levels of competences to build on are present at the time of application and a similar test like the GAP-test might be a helpful selection tool for medical students. Additionally, medical curriculum planners might wish to check carefully whether the competences measured by the GAP-test are included sufficiently in undergraduate training to equip medical students for their postgraduate work with respect to patient safety.

## Conclusions

Advanced undergraduate medical students show high scores for the competence domain adherence to procedures, which might be associated with the prediction of their successful performance in medical school as has been shown for conscientiousness. With respect to the competence domain awareness, which is important for clinical reasoning and patient safety, medical students do not reach the entrance level for flight school. Whether the competence of awareness should be included in medical students’ selection tests or whether specific training units for awareness need to be included in the undergraduate medical curriculum should be in the focus of further investigations. The domains of teamwork and leadership could also be improved and might be addressed in a more pronounced way during undergraduate medical training.

## Abbreviations

AP: adherence to procedures
AW: awareness
CM: communication
DLR: German Aerospace Center
GAP: Group Assessment Performance
LS: leadership
NEO-FFI: Neuroticism-Extraversion-Openness Five-Factor Inventory
OSCE: objective structured clinical examination
PY: final year
S10: semester 10
SR: stress resistance
TW: teamwork
VI: vertically integrated
